# Tranquillized but at risk: the conservation cost of rhino capture

**DOI:** 10.1093/conphys/coaf053

**Published:** 2025-07-23

**Authors:** Jennifer M Cocciardi

**Affiliations:** Department of Biology, University of Mississippi, Oxford, MS 38655, USA

Sedation is an essential tool in the fight to save white rhinos (*Ceratotherium simum ssp. Simum)*, but it may come with unintended risks. Conservationists rely on powerful tranquillisers to safely immobilize these vulnerable animals—but new research by an international team of researchers (Boesch *et al.,* 2025) reveals that one commonly used drug, etorphine, may come with serious physiological side effects.

Across the southern Africa, conservationists use a potent opioid, etorphine, to sedate white rhinos (Fig. 1)—not just for relocation, but to deter poaching through dehorning, collecting biological samples, and delivering veterinary care. Yet, this drug can trigger a stress response that causes dangerously low blood-oxygen levels, a high heart rate, and intense metabolic strain. In doing so, it may cause unintentional morbidity and mortality. The promising news? An extra dose of butorphanol, a medication that partially blocks the effects of strong opioids, appears to reduce some of these side-effects and can offer a safer approach to rhino sedation.

To investigate how etorphine affects rhino physiology—and whether butorphanol can ease it’s negative effects—Boesch and colleagues conducted a paired experiment on six subadult male white rhinoceroses. Each animal received two treatments in random order, two weeks apart: etorphine followed by saline and etorphine followed by butorphanol. The moment each rhino became horizontal was marked as time zero, after which researchers fitted the animals with monitoring equipment. At 30-, 40- and 50-minute-post-immobilization, researchers measured a suite of physiological variables including blood oxygen and carbon dioxide levels**,** heart rate, respiratory rate, blood pressure, metabolic rate and plasma catecholamine concentrations**.**

**Figure 1:**
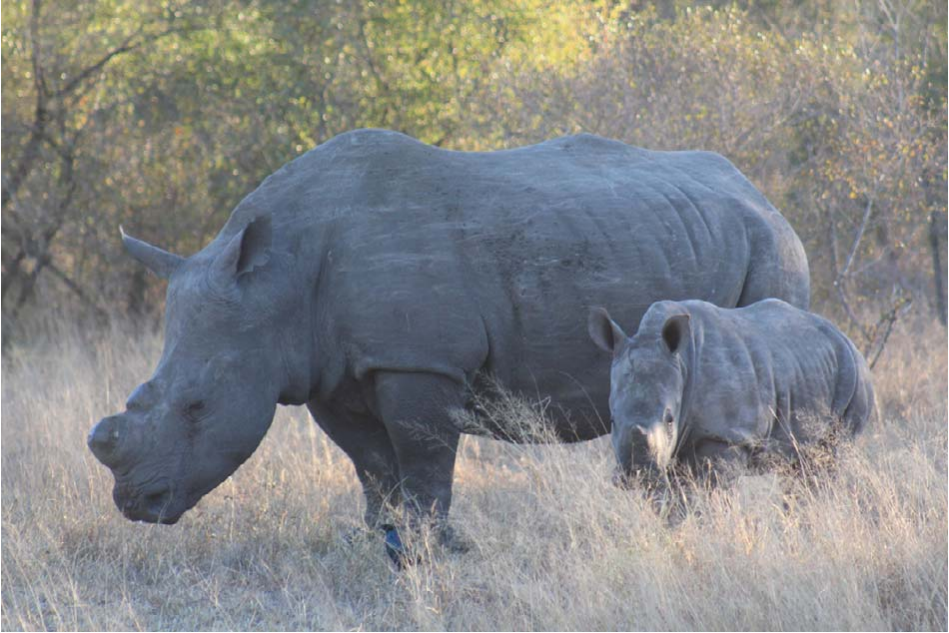
Adult female white rhinoceros and calf. Image credit: Jordyn Boesch

The data revealed important differences in how individuals responded to each drug combination. When given butorphanol after etorphine, blood oxygen levels increased and oxygen consumption, heart rate, and pulmonary pressures all decreased—indicating that butorphanol helps to ease metabolic stress and improves oxygenation during immobilization. Although some signs of stress remained (like elevated noradrenaline levels), the overall response indicated that physiological stress was moderated. This suggests that butorphanol helps to modulate the ‘fight-or-flight’ response caused by etorphine without reversing the effects of immobilization.

These findings have important implications for rhino conservation. As poaching and habitat loss continue to threaten white rhinoceros’ populations, safe and effective immobilization is essential for successful conservation actions. This study underscores the need to examine adverse effects of immobilization to ensure we are using the safest possible methods. By studying how immobilizing drugs affect rhino physiology, we gain a more complete understanding of their effects and can use this knowledge to improve immobilization protocols. With effective and safe drug combinations, conservation teams can keep doing essential work without putting rhinos at unnecessary risk.

## Notes

**Editor:** Ashleigh Donaldson
